# Supplementary material for: Multicommodity routing optimization for engineering networks
Source: Sci Rep. 2022 May 6;12:7474. doi: 10.1038/s41598-022-11348-9 (PMC9076927; doi:10.1038/s41598-022-11348-9)
Supplement: Supplementary file 1 — Supplementary Information. [file 41598_2022_11348_MOESM1_ESM.pdf]

Multicommodity routing optimization for engineering networks: Supplementary Information (SI)

Alessandro Lonardi[1,*], Mario Putti[2], and Caterina De Bacco[1]

[1]Max Planck Institute for Intelligent Systems, Cyber Valley, Tübingen 72076, Germany
[2]Department of Mathematics “Tullio Levi-Civita”, University of Padua, Via Trieste 63, Padua, Italy
[*]alessandro.lonardi@tuebingen.mpg.de

1 Listing of important variables

In order to facilitate readability, in Table 1 we list some of the main parameters of our model.

Variable	Dimension	Definition	Interpretation
$G(V,E)$	—	—	Network, V = set of nodes, E = set of edges
$\ell=\{\ell_e\}$	$\lvert E\rvert$	—	Euclidian length of the edges
$B=\{B_{ve}\}$	$\lvert V\rvert\times\lvert E\rvert$	—	Signed incidence matrix of G
M	Scalar	—	Number of commodities
$S=\{S_v^i\}$	$\lvert V\rvert\times M$	—	Mass matrix containing in/outflows of passengers
$\mu=\{\mu_e\}$	$\lvert E\rvert$	—	Edge conductivities
$L=\{L_{uv}\}$	$\lvert V\rvert\times\lvert V\rvert$	$L_{uv}:=\sum_e(\mu_e/\ell_e)B_{ue}B_{ve}$	Weighted Laplacian matrix of G
β	Scalar	—	Regulatory parameter for traffic congestion
$p=\{p_v\}$	$\lvert V\rvert$	—	Pressure potentials on nodes
$F=\{F_e^i\}$	$\lvert E\rvert\times M$	$F_e^i:=\mu_e(p_u^i-p_v^i)/\ell_e$	Fluxes on edges generated by the commodities
$f(\cdot)$	Scalar function	$f(\cdot)=\lvert\lvert\cdot\rvert\rvert_1^2$; $f(\cdot)=\lvert\lvert\cdot\rvert\rvert_2^2$	Response function for fluxes coupling
J	Scalar function	$J:=(1/2)\sum_e\ell_e f(F_e)/\mu_e$	Dissipation cost
W	Scalar function	$W:=(\sum_e\ell_e\mu_e^{\gamma})/2\gamma$	Infrastructure cost
J_Γ	Scalar function	$J_\Gamma:=\sum_e\ell_e f(F_e)^\Gamma$	Dissipation cost, unconstrained minimization problem
$\mathscr{L}_\beta$	Scalar function	$\mathscr{L}_\beta:=\frac{1}{2}\sum_{i,v}p_v^iS_v^i+\frac{1}{2(2-\beta)}\sum_e\ell_e\mu_e^{2-\beta}$	Lyapunov functional
γ,δ,Γ	Scalars	$\gamma:=2-\beta$; $\delta:=1/(3-\beta)$; $\Gamma:=(2-\beta)/(3-\beta)$	Auxiliary critical exponents
ρ	Scalar	—	Parameter for mass matrix smoothing

Table 1. Comprehensive listing of the main parameters and variables used.

2 Optimal transport networks

In the panels in Fig. 1 and Fig. 2 we show the optimal transport networks for different configuration of the input forcings $S(\rho)$. In detail, we display the results for $\rho=0.5$ (Fig. 1), and those for $\rho=1.0$ (Fig. 2). Looking at the rightmost networks ($\beta=1.5$) of Fig. 1a and Fig. 2a one can observe a clear tendency of the 1-norm dynamics to concentrate traffic more than the 2-norm one. This trend reflects on the sorted distributions plotted in Fig. 1c and Fig. 2c, where the fluxes are more fat-tailed and homogeneous for the 2-norm. Notably, the effect becomes starker the more the input inflows of passengers distribute uniformly on the nodes, i.e. increasing ρ.

3 Network resistance to failures: 2-norm dynamics

In Fig. 3 we reproduce the experiments designed to test the resilience of optimal networks to node failures. Overall, results are similar to those in the main text, it is worth mentioning how the Gini coefficient values Fig. 3c are higher than the correspondent ones for the 1-norm, symptom of the tendency of the latter forcing function to aggregate traffic. Another implication of this effect is that the Gini coefficient values for the 2-norm tend to separate more for higher β than those of the 1-norm. In fact, the latter tend to be overlapped, regardless of the number of failures, on a larger portion of the x-axis (where $\beta>1$).

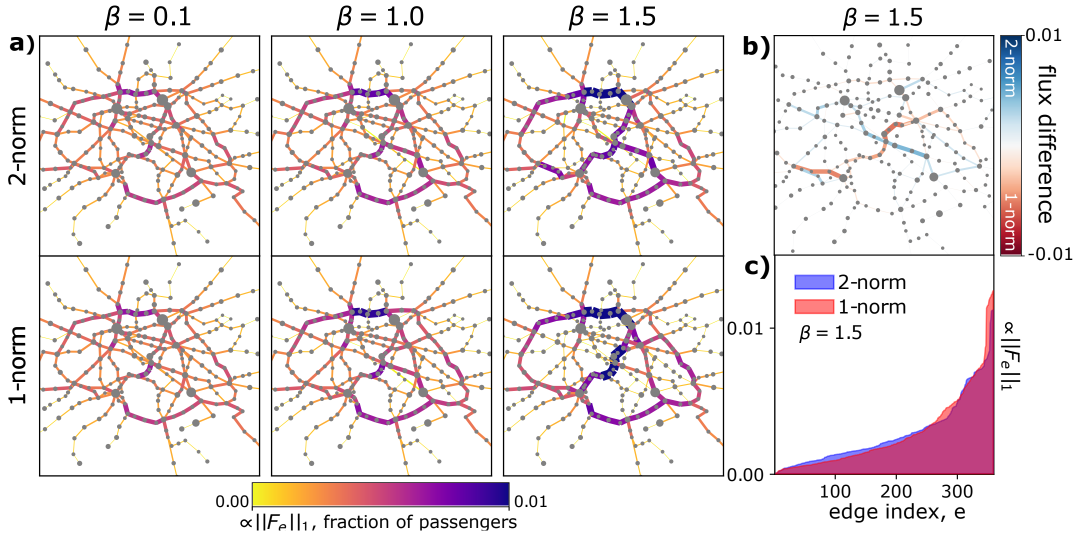

Figure 1. Optimal transport networks panel with forcing $S(\rho = 0.5)$. For a detailed description of the subplots one can refer to Fig. 2 in the main text.

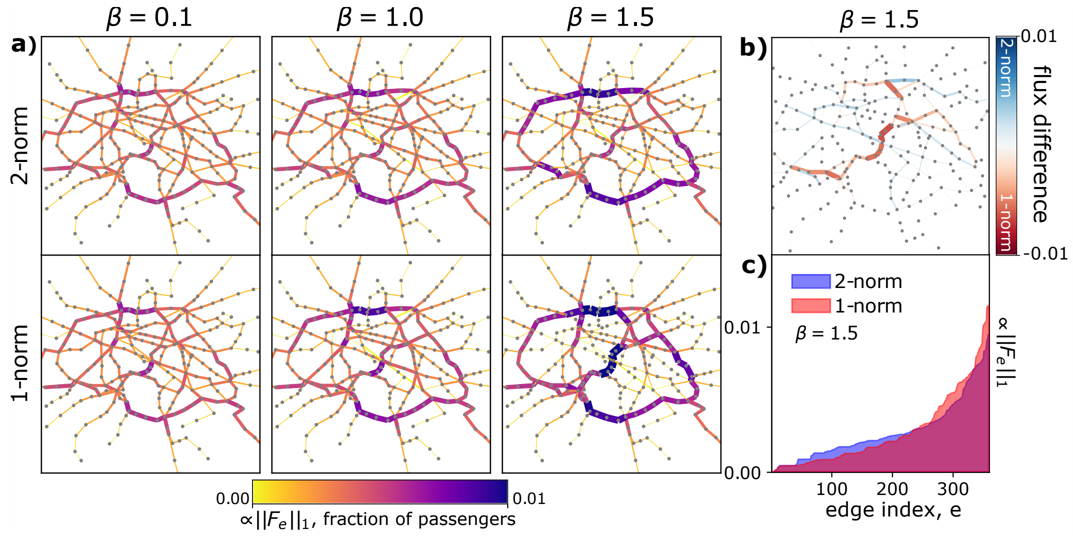

Figure 2. Optimal transport networks panel with forcing $S(\rho = 1.0)$. For a detailed description of the subplots one can refer to Fig. 2 in the main text.

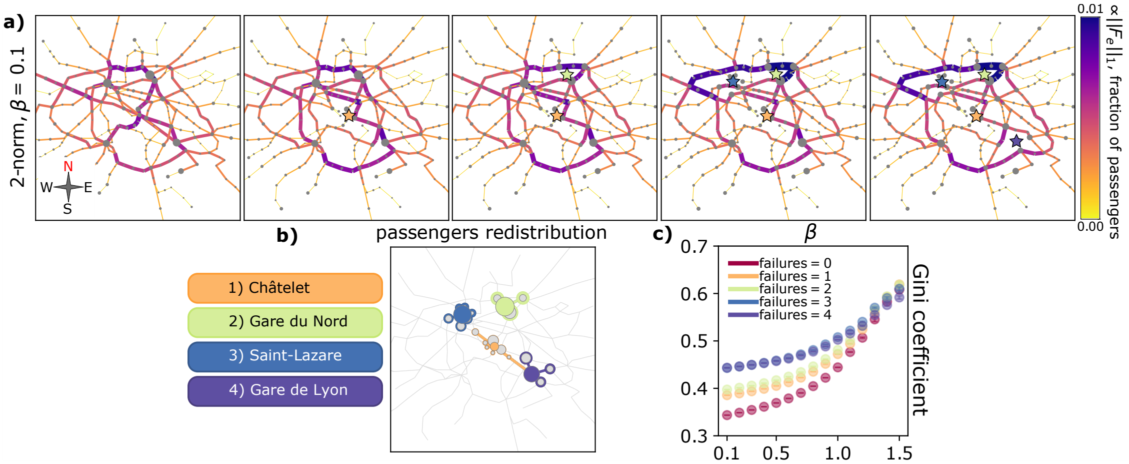

Figure 3. Traffic rerouting after network structural failures (2-norm dynamics). For a detailed description of the subplots one can refer to Fig. 5 in the main text.
